# Commonalities and Divergences in the Cognitive Profiles of Autism and Dementia: Protocol for a Narrative Literature Review

**DOI:** 10.2196/82349

**Published:** 2026-05-12

**Authors:** Lynsey Stewart, William J Mcgeown, Jonathan Delafield-Butt, Mario A Parra

**Affiliations:** 1 Department of Psychological Sciences and Health, University of Strathclyde, Graham Hills Building 40 George Street, Glasgow, G1 1QE, United Kingdom, 44 7921462396; 2 Laboratory for Innovation in Autism, University of Strathclyde, Glasgow, United Kingdom; 3 Strathclyde Institute of Education, University of Strathclyde, Glasgow, United Kingdom

**Keywords:** autism, dementia, cognition, aging, healthy aging, neurodiversity, Alzheimer disease

## Abstract

**Background:**

Literature has shown an increase in research relating to autism and aging, and more specifically, autism and healthy aging. Within the literature, there is a clear lack of knowledge and understanding of the impacts of dementia and autism as co-occurring experiences. More specifically, there is a lack of clinical knowledge about the ways in which the cognitive profiles of autism and dementia do and do not overlap, and how this overlap might affect dementia assessments for this population. This is likely to result in challenges with diagnosis and may lead to misdiagnosis or a lack of diagnosis for the autistic population.

**Objective:**

This paper reports on a narrative literature review that addresses the lack of understanding of potential similarities and differences in the cognitive profiles of autistic people and patients with dementia. It aims to identify the cognitive functions that are sensitive only to dementia and are less likely to be sensitive to autism. This will allow for a deeper understanding of how to grant sensitivity and specificity in the assessment of dementia within the autistic population.

**Methods:**

The narrative literature review relies on existing guidelines (eg, PRISMA [Preferred Reporting Items for Systematic Reviews and Meta-Analyses]) to inform a newly developed tool, namely the Quality of Evidence Tool (QoET), used to assess publications identified in relevant databases (eg, PubMed, PsycINFO, and others) that report on cognitive functions in autism. Four main cognitive domains were chosen: memory, executive function, attention, and language, as these are the most commonly impaired in people with dementia due to Alzheimer disease and are known to be affected by autism. The QoET was used to select publications that informed us of cognitive functions that would meet our aim, that is, functions affected by dementia and not by autism.

**Results:**

This narrative review will identify cognitive domains and functions affected by dementia, not autism, and the assessment tools that yield these findings. It is expected that the rigorous methodological approach devised will shed light on how existing cognitive assessments should be used to increase the specificity of dementia risk detection among autistic individuals, thereby addressing early concerns raised by individuals or health care practitioners. It is expected that the review will be ready for publication by December 2026. As of April 2026, memory and executive function have been drafted.

**Conclusions:**

This narrative review does not seek to give a definitive answer on the cognitive domain, function, and outcome measures or tests used in dementia detection, which can help detect risk in autistic individuals. Instead, it aims to provide a starting point, to pragmatically and efficiently explore and synthesize the broad evidence base, to inform future work and potentially highlight topics (eg, cognitive functions of interest) on which future systematic reviews could specifically focus.

## Introduction

### A Note on Language

There has been a vast amount of research exploring the significance of language for the autistic and neurodivergent community [1]. Research shows that the majority of autistic people prefer identity-first language [2]. Recently, there has been a move to avoid deficit-based language, such as “impairment” or “disorder.” Evidence shows that referring to “difference” is more accurate and inclusive [3]. Literature is beginning to report that the autistic experience is often misunderstood by societal standards and differences in cognition have previously been classed as deficits [4]. The language in this paper has been designed to reflect “difference” rather than “deficit” wherever possible.

It is also recognized that language about the specific symptoms of dementia tends to be deficit- or impairment-based due to the nature of dementia being a neurodegenerative disease, defined largely by impairments [5].

There is a distinction in this paper between the cognitive differences experienced by autistic people and the cognitive decline or impairments caused by dementia. To keep this distinction clear, the paper will mainly refer to differences and/or impairments. Where one is specifically meant or being referred to, this will be stated. When “impairment” or “deficit” is used, it will only be when referring to objective evidence of lower performance in a particular function.

### Background

Research is beginning to report more on the experiences of older autistic people [6]. The topic of autism and aging has recently been highlighted as a priority [7]. Stringfellow et al [6] reported on the increase in literature over the last 5 years, but noted the sparse evidence in relation to healthy aging for autistic adults. A preliminary exploration of the literature highlighted that, within this topic, there is very little research focusing on the impacts of dementia on autistic people or on the impact of autism on dementia. The lack of understanding of the relationship between autism and dementia needs to be addressed, as do the reasons for the lack of data. This may be due to reasons such as small sample sizes, inappropriate diagnostic systems, or a lack of understanding and awareness in clinics, but further evidence is needed. At least 1% of the general population is autistic [8], and it is estimated that approximately 93,000 people in Scotland have dementia [9]. The intersection of these 2 conditions needs to be better understood [10], as does the possibility that there are autistic people who also have progressive cognitive impairment likely linked to dementia risk, and who are currently undiagnosed and consequently lacking adequate support. There is recognition in the literature of this gap and the challenges it is likely to cause for the autistic population [11].

The literature that does explore autism and dementia as co-occurring conditions is divided in relation to prevalence rates. Vivanti et al [12] found that autistic people were more at risk of developing early-onset dementia. Mohammadian et al [13] found an 18.5% prevalence of autism symptoms among the 65 patients with dementia they screened. Harker et al [14] found that autistic middle-aged adults appear to be more likely to receive a diagnosis of Alzheimer disease (AD) or other dementias than nonautistic people. The authors reported that there is a higher likelihood of a specific gene, APOE4 (apolipoprotein E4), in autistic middle-aged adults, which increases the risk of developing AD. Conversely, in a study analyzing data from over 1700 individuals with autism listed as a cause of death [15], it was suggested that the autistic population is less likely to develop dementia.

The unfavorable status quo is characterized by a lack of research on the shared and distinct cognitive profiles of autism and dementia, which undermines the early and accurate detection of the latter condition in the former population. Key factors underpinning this are (1) a lack of understanding of autism in aging among clinicians [12], including how different types of dementia may manifest in older autistic adults; (2) contributing factors in diagnostic processes leading to a putative large population of undiagnosed autistic adults in their 50s and older [10]; and (3) limitations in the available knowledge on the cognitive functions that can distinguish dementia and autism-related cognitive profiles [15]. Additionally, there is a lack of normative data for neurodivergent populations on the existing cognitive tests used in dementia assessment [11]. This, again, may reduce their use and accuracy.

This review will focus on the third key factor mentioned above and aims to identify cognitive functions that are spared in autistic people but impaired in people with dementia, as well as identify the sources of commonalities and divergences. Dementia is a broad umbrella term that encompasses several diseases. Given the expansive scope of this narrative review, which covers many areas of cognition in both autism and AD, there is no scope within this project to move beyond the AD dementia type due to the depth and amount of literature that would be required to be reviewed. As existing literature shows that AD is the most common form of dementia and the one presenting a global challenge [16], this is a worthwhile starting point.

Current research has demonstrated the need for specific and bespoke assessment methods that consider the potential overlapping symptoms of autism and dementia [16]. It shows the higher risk of misdiagnosis when specialist screening tools are not used. Stringfellow et al [6] note the need for greater understanding of autistic cognition and aging to help mitigate the risk of conditions such as AD. Janecki et al [17] reported that knowledge of cognitive health and decline in the autistic population is lacking due to it being underresearched, and this is supported by a study by O’Donald et al [11].

By focusing on AD, this review will be the first step in the characterization of the overlapping and distinctive cognitive features of autism and dementia, particularly those caused by AD. This new knowledge will provide a foundational basis for the subsequent selection or creation of psychological assessments that are suitable and optimized for identifying progressive cognitive decline and dementia in autistic people. These knowledge gains and innovations in instrumentation, bespoke for older autistic people, will facilitate improved clinical care and support for advances in health and mental health outcomes.

### Aims and Objectives

This narrative literature review will identify profiles of cognitive impairments that can reliably inform the development of assessments for the presence of dementia or the risk of developing this condition within an underrepresented and understudied aging (midlife onward) autistic population.

To achieve this aim, the cognitive impairments that exclusively and more frequently affect people with or at-risk of dementia must be taken into consideration. However, this poses challenges, as many of the cognitive impairments and differences seen in dementia and the risk of dementia can also be observed in autistic people, though, perhaps to a lesser extent [15]. To understand and characterize the combination of autism and dementia, it is important to have a reliable, evidence-based cognitive profile of autism in people with or at risk of dementia, which helps identify those who have embarked on an abnormal aging path.

The primary cognitive functions affected by AD in its typical presentation variants are short- and long-term memory, executive function, language, and concentration and attention span [18]. The extant literature raises the possibility that the cognitive profiles of autism and dementia overlap [19], which suggests that traditional assessments for dementia may not be effective for use with autistic people due to their lack of specificity.

For example, a patient undergoing a dementia assessment may be assessed for changes to long-term verbal memory. If this patient was autistic and scored poorly on this task, it might be because this function was already sensitive to autism. Therefore, the results of that task might not be an accurate indication of cognitive decline due to dementia, as they may not be related to dementia at all or may only be partially related. We can see an example of this in visual short-term memory binding [20]. Based on evidence that this function is impaired in the population with AD but not in normal aging or in populations without AD, Parra et al [21] developed tests to assess this function in patients with AD. These tests have proven to be a preclinical marker for AD [22]. However, evidence suggests that binding is impaired in the autistic population [23]. Therefore, a test designed to assess this function would not be able to determine whether the impairments were due to the onset of AD or were due to the existing autistic cognitive profile.

Similarly, a serious reduction in that function might not be evident if differences in function already exist and are not accounted for at the start of the assessment. It is recognized that standard practice includes taking a baseline from a patient and working from there; however, it may be the case that a baseline is not recorded early in the individual’s aging process. For example, if an autistic person had a particular strength in a certain function (eg, long-term verbal memory) due to the cognitive effects of autism, they might originally score highly on a particular test designed to measure this function. If a baseline had not been taken, a reduction in this function could occur further down the line, but performance might still not meet a threshold for abnormality based on the neurotypical average, resulting in the reduction in functioning being missed. There is the added factor of how these functions are assessed, as specific assessments, processes, materials, and applications may not take into account autistic cognition. This could lead to an indicator of reduced cognitive function being missed, amplified, or misunderstood. Any of these outcomes could result in a misdiagnosis. There is evidence that the misdiagnosis of dementia results in poorer outcomes and life experiences for those affected [24].

We can see similar obstacles in the field of aging and dementia. The assessment of cognitive functions, which show an age-related decline and are further impacted by some forms of dementia, has proved challenging [18]. A good test for dementia needs to be both sensitive and specific [25]. For example, the Visual Short-Term Memory Binding test developed by Parra et al [20] has proved insensitive to normal aging and has aided the detection of AD in the preclinical stages [26]. This is a good example of a bespoke detection method designed to be sensitive to an aspect of cognition of which decline is known to be indicative of disease. This narrative literature review will aim to support the development of a bespoke detection method designed to be sensitive to specific cognitive features that indicate an autistic patient is at-risk of or already living with dementia.

Consideration needs to be given to the heterogeneous nature of autism. Research shows there is a wide range of experiences of autism, from having high support needs to having lower support needs [27]. This heterogeneity will also impact assessment outcomes and will be addressed in this review.

To decrease the likelihood of a misdiagnosis, a specific assessment toolkit that considers and mitigates against a modified cognitive profile due to autism could be a crucial factor in improving dementia detection and diagnosis in autistic people. Such an assessment tool would focus only on the cognitive functions that are likely to be relatively insensitive to autism, but much more likely to be sensitive to dementia. Isolating these functions will provide a focus and structure on which to build the assessment. It is crucial that this is based on high-quality theory and evidence. This narrative literature review will develop a framework that will result in a series of visual mind maps depicting the most promising cognitive domains for use within a bespoke assessment toolkit.

There is no current empirical evidence that goes into the specific details of identifying such a profile. However, literature is available in relation to both as single conditions. This literature will form the basis of the evidence for the cognitive profile maps. The maps will break down each of the cognitive functions and evaluate sensitivities.

To provide a robust and reliable evidence base, it is necessary to explore each of the cognitive functions that form part of a dementia assessment. There is a wide range of cognitive domains that could be studied; however, the scope of this paper does not allow for such a wide exploration. To this end, 4 domains were chosen a priori based on existing literature as the most common ones examined in a dementia assessment.

The literature supports the most commonly affected domains as memory [28-30], attention [31-33], language [34-36], and executive function [37-39]. Therefore, this review will focus on the broad cognitive domains of memory, attention, language, and executive function.

It is recognized that there is emerging evidence suggesting that other domains may also be appropriate, such as social cognition, motor control, and reasoning; however, evidence for these is still in the early stages and is therefore less reliable [40].

Each domain comprises different functions. There may be some functions within a domain which would give promising results by being sensitive only to dementia and relatively insensitive to autism, while others are sensitive to both. Therefore, a variety of specific functions within each domain will be assessed during the review.

This narrative literature review aims to understand how cognitive assessments to support the diagnosis of dementia would work. This includes (1) areas of cognition that are sensitive to autism and dementia; (2) areas of cognition that are relatively insensitive to autism but sensitive to dementia, even from its early stages; and (3) the most commonly used assessments to test the functions identified via objective 2.

## Methods

### Design

A critical appraisal of the literature is being used to identify the most promising domains. To ensure the validity and reliability of the results, a Quality of Evidence Tool (QoET) has been created to work alongside the inclusion criteria.

The evidence base will be formed using a critical appraisal of existing literature. To review the literature in the most robust way, a customized tool has been developed to apply a quality check to the literature, based on existing tools—for example, the Joanna Briggs Institute Critical Appraisal Tools [41]—and guidelines and frameworks to guide reviews, such as PRISMA (Preferred Reporting Items for Systematic Reviews and Meta-Analyses) [42] and Cochrane [43]. This tool is designed to take into account the heterogeneity of the autistic profile, which existing tools and frameworks do not account for [44].

Due to the wide scope and heterogeneity of autism, and the breadth of cognitive function investigated, it would be unfeasible at this stage to carry out a systematic review that is representative of and equal for every domain. To that end, a narrative review will be carried out. Traditionally, narrative reviews are best suited for providing a broad overview, state-of-the-art approaches, and theory-building. We have followed best practices by undertaking purposive research, iterative reading, and thematic organization, following an approach akin to that proposed by Ferrari [45].

While there is no scope to carry out a full systematic literature review for the number of cognitive domains and functions being evaluated, the QoET is intended to provide objective evidence to support decision-making. The outcome of this high-level review of the literature can inform future systematic reviews because it will provide a more granular review of the literature so that systematic approaches can be implemented in the future (eg, approaches that can then target specific cognitive functions of interest). The narrative literature review will aim to (1) identify relevant studies and domains, (2) justify study selection through the use of the QoET, (3) extract the data, and (4) summarize and evaluate the findings and results.

These tools (PRISMA and Cochrane) will not be directly applied to studies once identified, but they will help select suitable papers via the QoET. Drawing on them will allow the review to follow the approach these frameworks recommend regarding the selection of studies. The QoET is being used to give the best chance of providing evidence to address the question posed by this narrative review. However, the QoET does not allow a pathway to a systematic approach but provides a level of confidence that the studies being selected do provide the best available evidence. This narrative review will follow narrative guidance, such as Ferrari [45], in terms of structure and processes.

### Purpose

The purpose of this review is to identify the most promising cognitive functions and tests within the broader domains. Within each cognitive domain—for example, memory—there are subdomains, such as short-term memory, and tasks and tools used to assess these subdomains. For the purposes of this review, we are primarily interested in identifying and evaluating the subdomains and relevant outcome measures or tests.

When identifying optimal cognitive subdomains for use in a customized assessment package, the QoET will be used to draw evidence of consistency to ensure there is logical reasoning behind which subdomains may be selected. The rationale behind this is that subdomains are more likely to provide information on the function or task to be considered than the domains.

### Population

There will be 2 primary populations of interest. The first will be individuals diagnosed with autism, and the second will be patients with dementia. The review will focus on papers that report on the cognitive functions of adults diagnosed with autism or dementia. Given the broad scope of this review, it is not currently feasible to extend the analysis beyond adult populations. As this review is intended to provide an initial foundation for future research, the decision to focus exclusively on adults reflects the reality that dementia predominantly affects individuals over the age of 50 years [46]. At this stage, including studies involving children would be unlikely to yield insights relevant to the primary aims of the review and risks weakening the focus. The purpose of the papers that focus on the population of those diagnosed with dementia is to confirm that the domains are affected. The purpose of the papers that focus on the population of those diagnosed with autism will be to map out the evidence for where sensitivities (eg, differences or impairments) in those domains do or do not lie.

### Study Selection and Quality of Evidence Tool

The literature on dementia will be used to identify studies that support the view about the main cognitive functions affected by these disorders, which are considered in this review. This literature is vast and longstanding [5]. The literature on autism and cognition is a newer area of research and requires further exploration. Therefore, for this set of literature, the quality of evidence tool will be applied to assist in evaluating which cognitive subdomains appear to be affected by autism and which may be most appropriate to focus on for the assessment of dementia (or risk of dementia).

### Search Strategy

The narrative review will not adopt a formal systematic review methodology but will implement a structured and transparent search strategy to identify and synthesize relevant literature. The full review will be organized by cognitive domain, with each domain presented in a dedicated subsection. Each subsection will describe the specific search procedures used to interrogate the existing literature. Search terms were kept as broad as possible regarding the nature of the autistic populations and methodologies used, as limiting them would also limit the scope, interpretability, and applicability of the results to the investigated problem. Dementia is expected to affect the full spectrum of autistic people.

To illustrate the planned approach, the example below presents examples of a search strategy and a search string that will be used to identify studies related to the memory domain.

Searches will be conducted across major academic databases, including APA PsycINFO, PubMed, Web of Science, and Jisc Library Hub Discover.

In addition, citation searching from relevant studies will be used to identify sources that may not have been retrieved during the original search (Textbox 1).

The timeline for the search strategy includes the time period from June 1, 2024, to March 1, 2026.

Textbox 1. **Search string for memory.**

(autism OR ASD OR ASC OR autistic OR autis*)

AND

(cognition OR cognitive OR cogniti* OR memory OR “long-term memory” OR “short-term memory”

OR “visual memory” OR “visuospatial memory” OR “verbal memory” OR “episodic memory”

OR “autobiographical memory”)

AND

(impairment OR impairments OR difference OR differences OR profile OR profiles

OR deficit OR deficits OR disruption OR disruptions

### Eligibility Criteria

A 2-step abstract and title screening will be conducted following the eligibility criteria. Inclusion criteria for autism papers were as follows: (1) compares autism with typically developing adults, (2) published in a peer-reviewed journal, (3) published in English, and (4) the paper compares cognitive abilities between the groups.

The search has not been limited to specific years. As a broad narrative review addressing a wide knowledge gap, the search has been kept as broad as possible. It is likely that the representativeness of the various cognitive domains explored in the autism literature will vary greatly, and limiting the search time window would further impact such representativeness.

Non-English publications will be excluded due to the practical constraints of translation and the predominance of English within scientific research literature [47]. Gray literature, such as theses, reports, and conference abstracts, will not be included, as the review aims to synthesize evidence from peer-reviewed sources with consistent methodological standards. It is acknowledged that this may be a limitation of the study, and this should be considered for future research.

Where available, meta-analyses and systematic reviews will be prioritized, as these will provide a synthesized overview of the literature based on a broader analysis rather than individual studies. All references will be collated into a single reference manager (EndNote 20; Clarivate), where duplicate entries will be removed.

### Data Extraction

Data extraction will be conducted using a structured Microsoft Excel form developed specifically for this review, including fields for study characteristics, cognitive domains, effect sizes, and duplication checks. This will ensure consistency and transparency across all included studies.

### Avoidance of Duplication

When systematic reviews or meta-analyses are included, the references are checked both manually by the researcher and using artificial intelligence tools to identify duplicates. If duplication is identified, the primary and secondary sources will be identified. The primary source will be used for inclusion. The secondary sources will be removed, but the references will be kept for context or confidence statement.

### Piloting of the Tool

A pilot of the quality of evidence tool with 2 independent researchers, applying it to a selected group of papers per cognitive domain, will be undertaken. Each reviewer will be given papers selected at random from each of the domains and asked to apply the QoET to them. These scores will be compared to those given by the lead researcher. Interrater reliability will be assessed using intraclass correlation coefficients (ICCs). The lead researcher will then conduct a further review of each of the papers included.

### Worked Example

**Figure 1.**
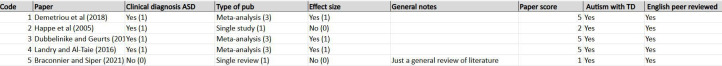
A worked example of how the Quality of Evidence Tool will be applied to papers. TD: typically developing.

Figure 1 shows a worked example of how the QoET is applied to papers.

Multimedia Appendix 1 shows the standard extraction table and gives examples of how this will be applied.

### Quality of Evidence Tool

#### Overview

The customized tool will use the literature to provide robust evidence by comparing the autistic population to the neurotypical population to show where there are and are not differences in cognition. The narrative literature review draws aspects from the Joanna Briggs Institute Critical Appraisal Tools [41], PRISMA guidelines [42], and the Cochrane Handbook [48] and incorporates new criteria specific to this project’s purposes. The narrative literature review is primarily interested in functions that are less likely to be affected by or sensitive to autism. This is represented in existing literature by small effect sizes or the reporting of small (or no) impairments. A domain that has medium to large effect sizes or is reported to have medium to large impairments will be more sensitive to or affected by autism and, therefore, will be less suitable. When interpreting effect sizes, the review draws on established guidance such as Cohen *d* to classify the magnitude of effects. This supports consistent interpretation across studies while maintaining the narrative format. Once the small effect sizes or reporting of small impairments in autism have been established, we will corroborate whether there is a large or significant effect size in dementia. This will be done using the existing dementia papers with the following inclusion criteria: (1) compares dementia (focusing on AD) with typically developing adults, (2) published in a peer-reviewed journal, (3) published in English, and (4) compares cognitive abilities between the groups.

**Figure 2.**
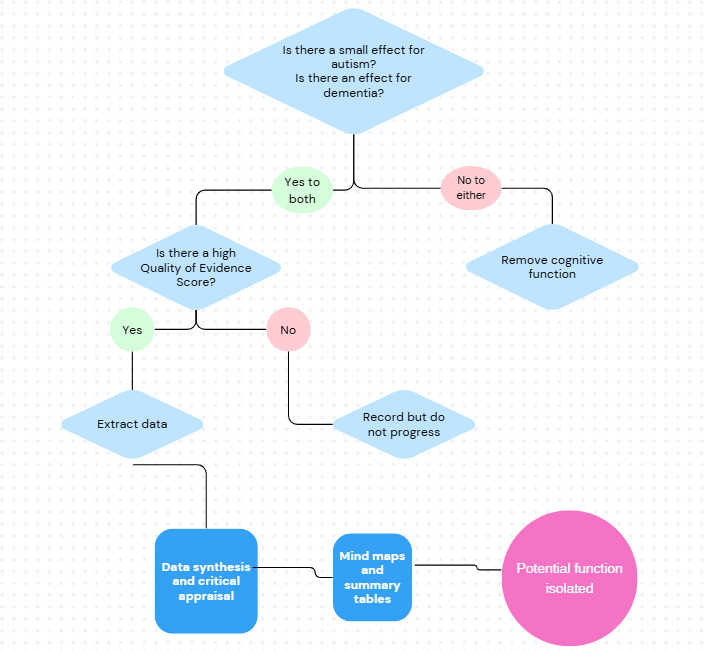
Flowchart for applying the Quality of Evidence Tool and extracting data for cognitive functions.

To narrow down the vast literature search results, a flow system is used to identify the cognitive functions that do not have a reported significant difference or a large or medium-to-large effect size in the autistic population. If a function is reported as having a small effect size (in relation to autism) or an insignificant difference (autism compared to the typical population), then the next stage of the tool will be applied. If the function is reported to have a medium or large effect size or a significant difference, then it will not be explored further. Similarly, if the literature suggests that the function is not sensitive to dementia, it will also not be explored further. Therefore, only functions that have reported small effect sizes for autism and evidenced effects for dementia will be explored. There is scope for this to be updated in the future to cover additional literature, and the tools may be reapplied, modified, or updated. Figure 2 displays how this will be done.

Once the relevant functions have been identified, the QoET will then be applied. The QoET is broken down into 2 sections. One focuses on the evidence to support the specific cognitive function, and the other focuses on the reliability of each publication or paper (see “Calculation of Paper Scores” and “Calculation of Cognitive Function Scores” sections). These 2 scores are then added together to form a final score and rating for that specific cognitive domain. The functions with a “strong” rating will be the ones that are isolated as having potential for assessment.

#### Calculation of Paper Scores

This is designed to assess the quality of the evidence in terms of the validity and reliability of the evidence. This score will help mitigate against a function being identified as potentially useful when the evidence to support it is weak or unsubstantiated. This score will also help ensure that any papers included in the review have a consistent set of reliable and robust methods.

Considerations for this score include the inclusion of clinical diagnosis, type of publication, inclusion of effect size, and provision of inclusion criteria.

#### Calculation of Cognitive Function Scores

This is designed to assess the reliability and consistency of reporting. This score will help mitigate against a function being identified as potentially useful if, for example, it only has a single study with a small sample size to support the theory that it is or is not sensitive to autism. This score will help ensure that any functions identified have strong, reliable, consistent evidence to support their inclusion. Considerations in this score include consistency of reporting (ie, there is strong consistency in results across papers), the number of papers reporting on this domain, and the number of studies included in a review.

#### Scoring System

It is acknowledged that some of the weights assigned to the QoET parameters are arbitrary. It is aimed to recognize that an individual (primary) study would not be placed at the same level of strength as a systematic review. Dozois et al [49] proposed the hierarchy of research evidence related to clinical practice and placed systematic knowledge synthesis at the top of the hierarchy with primary research studies ranked as second and third, depending on their internal or external validity. It is intended that piloting the QoET to assess its interrater reliability and consistency will inform whether the proposed weighting yields reproducible, reliable results. By avoiding double-counting and adding pooled effects to individual effect sizes, it is intended to reduce the subjectivity of this approach. A detailed table of the scoring system is provided in Textbox 2.

Textbox 2. **Detailed overview of the QoET scoring system.**

Calculation of paper scores

- Do all autistic participants have a clinical diagnosis? (Not reported=0 points; no=0 points; yes=1 point)
- What is the type of publication? (Individual paper=1 point; systematic review=2 points; meta-analysis=3 points; systematic review + meta-analysis=3 points)
- Is effect size included (or mean and SD for each group)? (Not reported=0 points; no=0 points; yes=1 point)
- Does the review provide clear inclusion criteria? (Not included=0 points; no=0 points; yes=1 point)

Calculation of cognitive function scores

- Is there consistency across literature? (No consistency or not reported or 1 paper=0 points; up to 40% consistency=1 point; 41% consistency or higher=2 points; 100% consistency=3 points)
- If single studies: How many papers report on this function? (1 point per paper)
- If systematic reviews or meta-analyses: How many papers within each report were included with relation to this particular cognitive function? (Note each paper should only be given a score once, whether as an individual paper or as paper included in systematic reviews, to avoid duplication; 1 point per paper)

### Data Extraction

The final score ratings, along with evidence from the literature, will be used to identify how strong the evidence is that cognitive function is relatively insensitive to autism. Scores will be categorized to demonstrate whether there is weak evidence (20 and under), medium strength evidence (21-39), or strong evidence (40 and above).

### Critical Appraisal for Included Studies

The QoET is designed to support a critical appraisal of the included studies. Once the tool has been applied, a critical analysis of each paper will be carried out to ensure reliability and validity before a final decision on inclusion is made. Effect sizes will also be recorded so that a summary of these can help with any final judgments on what is an optimal assessment method to detect dementia risk in individuals with autism.

## Results

### Expected Results

This narrative review will determine the current studies exploring the cognitive domains and functions that are affected by dementia and not by autism. The results will generate summaries across domains, functions, and assessments, which can highlight which cognitive functions and assessment methods stand the best chance to detect dementia risk among autistic individuals in a way that cannot be accounted for by the latter condition. However, due to the heterogeneity that will underpin various aspects of this narrative review (diversity in autism and their comorbidities, different types of dementia and their phenotypes, different assessment tools, just to mention some relevant factors), it is expected that these results will pave the way toward a more controlled and targeted review, which can further distill the problems we are proposing here. The gap in the literature pertinent to this research question precludes the possibility of making predictions as to which function is anticipated to help tackle this outstanding problem. Nevertheless, it is expected that the rigorous methodological approach devised will shed light on how cognition should be assessed in autistic individuals whose risk profile for dementia could raise concerns either in the individual or among health care practitioners.

The results of this narrative review will be a novel addition to the literature as they will (1) clarify the relevance of considering the impact of neurodiversity on trajectories of neurodegeneration in aging, (2) provide the theory needed to support the development of novel assessments for autistic individuals, and (3) raise awareness among clinicians about the above.

### Presentation of Results

The extracted data will be presented in the form of diagrammatic mind maps. Mind maps for each cognitive domain will be provided for:

- Overview of the domain, subdomain, and outcome measures or tests
- Overview of the domain with small, medium, and large effect sizes
- Overview of the domain with small and medium effect sizes and QoET scores
- Overview of the promising domain, subdomain, and outcome measures or tests with QoET score

Additionally, an overall mind map will be included, covering all 4 of the cognitive domains reviewed and showing the promising domain, subdomain, and outcome measures or tests, for isolation.

**Figure 3.**
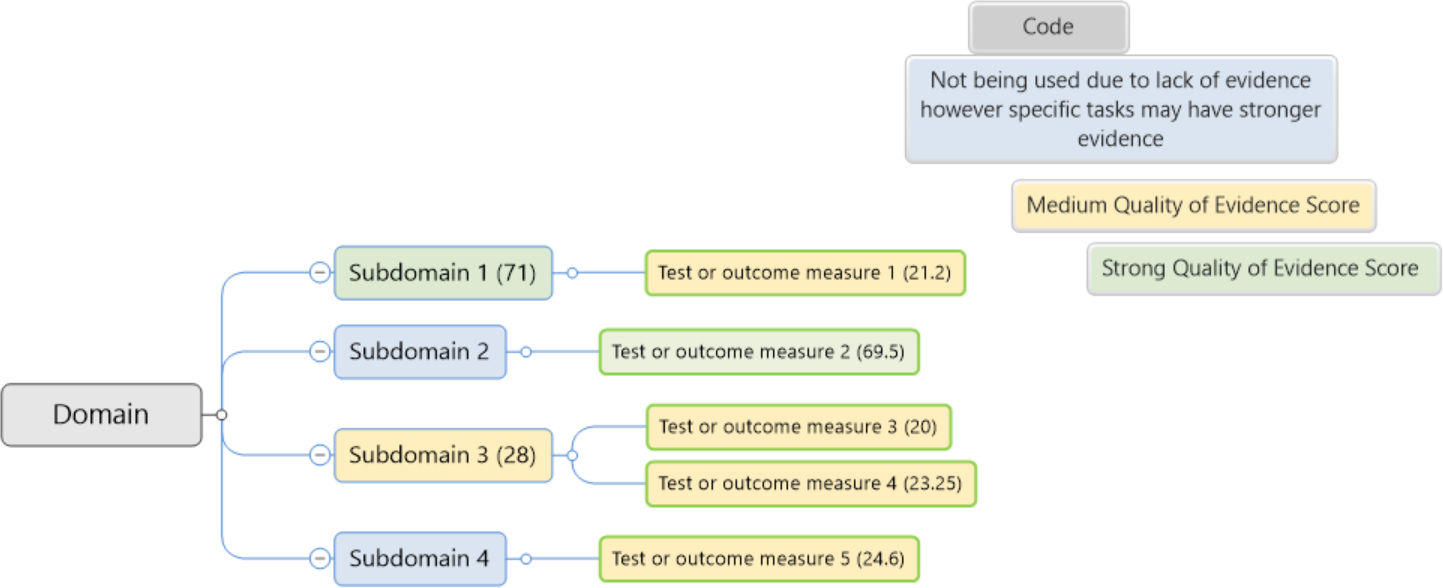
The conceptual structure of potential domains, subdomains, and outcome measures or tests and their strength of evidence scoring (strong or medium), with example placeholders to illustrate hierarchy and evidence mapping. The color code is listed in the top right corner.

An example of the mind map for promising domains and QoET score is given in Figure 3. Multimedia Appendix 1 will show full data for effect sizes (eg, mean, SD, ranges) and will include standard extraction data.

### Timeline

Table 1 shows the timeline in the form of a Gantt chart.

**Table 1.** Gantt chart of the project timeline.

Task	Completed January 2025	February 2026	March-May 2026	June-August 2026	September-November 2026	December 2026
Research questions or hypothesis	███████					
Development of search strategy	███████					
Development of QoET^a^	███████					
Search	███████ Memory Executive function	███████ Language Attention				
Addressing duplicates and double-counting			███████			
Applying the QoET		███████	███████			
Interrater reliability pilot			███████	███████		
Synthesis extraction and mind map building				███████	███████	███████
Generate final draft of the NR^b^						███████

a QoET: Quality of Evidence Tool.

b NR: narrative review.

## Discussion

### Summary

This narrative review does not seek to give a definitive answer on the cognitive domain>function>outcome measures, or tests used in dementia detection, which can help identify risk in autistic individuals. Instead, it aims to provide a starting point, to pragmatically and efficiently explore and synthesize the broad evidence base, to inform future work and potentially highlight topics (eg, cognitive functions of interest) on which future systematic reviews could specifically focus.

### Principle Findings

Each cognitive domain will have headings such as “Introduction,” “Methodology,” “Result,” and a “General Discussion.” The motivation for investigating each cognitive domain will be highlighted within each of these sections. These sections will also highlight what the key findings are for each cognitive domain and how those findings relate to the available knowledge, particularly in the field of dementia, and whether or not they have been considered in the autistic population.

### Comparison With Previous Work

To the best of our knowledge, this is the first narrative review that develops a screening tool (QoET) to identify suitable studies that meet criteria to address research questions that are outwith the scope of other existing tools (PRISMA and Cochrane) for systematic reviews. The aim of this tool is to pave the way toward the evidence needed to undertake a more refined and strategic review of valid studies (systematic reviews and meta-analyses). By addressing this gap, we hope to see future systematic literature reviews and meta-analyses supported by these more foundational steps, which aim to increase confidence and reliability in the identified evidence.

### Strengths and Limitations

To the best of our knowledge, this is the first study to explore commonalities and divergences in cognitive profiles in autism and dementia, with an emphasis on AD. To that end, we developed a novel tool that provides an innovative, bespoke, and practical way to explore the literature and extract high-quality evidence when a systematic review is not a feasible approach.

The lack of a full systematic review to attempt to capture all relevant literature could be a limitation; however, due to the broad range of cognitive functions and high number of available studies in combination, this is not achievable.

This review focuses on AD, the most common type of dementia. It is intended to be a starting point to help inform future work. It is acknowledged that results may differ for other types of dementia due to their distinct cognitive profiles, and that any potential bespoke tools developed outwith AD would need to consider this. This limitation should be addressed in future research, which should include the range of dementias and major disorders.

Similarly, the focus on the main cognitive domains affected by AD excludes other potentially promising domains, such as social cognition and motor skills, which are known to be affected in autism [50,51]. Future research should address this limitation by broadening the scope to include a wider range of cognitive domains beyond the 4 domains reviewed in this paper. In addition, future research should also consider the wider population beyond adults, as exploring developmental trajectories has been shown to provide a layer of insight into the aging population [52].

### Consideration for Future Work

There is not one single profile of autism, and each individual’s experience is unique [53]. This can result in varying levels of support needs, communication needs, and general life skills. These factors must be addressed when considering the modality of the assessments used. It may be that a bespoke tool is required to identify options for modality and specify the optimal circumstances for use. For example, for an autistic person who does not use verbal communication, a test modality that requires verbal answers would not be appropriate. Similarly, for an individual who requires high levels of support, an assessment that requires independent actions might not be suitable.

This review takes a very general approach to contribute to the knowledge base, distinguishing between the 2 cognitive profiles of autism and dementia. To this end, eligibility criteria have deliberately been kept broad to allow for as wide an understanding as possible. It is acknowledged that the clinical heterogeneity of autism, along with the wealth of comorbidities and other factors within the autistic population, is likely to impact cognition. Future research should explore more specific questions about the influence of these factors on cognition.

Contemporary autism research identifies the long-term effects of masking on autistic individuals [54]. It is recognized that masking can and does affect many aspects of an autistic person’s life. This may include certain aspects of cognitive function. To this end, future research should consider how masking might impact the effectiveness of the assessment tools identified in this review and explore how clinicians can adapt practices by both understanding and adapting for this.

### Impact

This review will provide a novel understanding of an underresearched topic. It will provide a robust evidence base to develop a bespoke assessment toolkit for use within the autistic community. The review will serve as a starting point for future research but, in itself, will give valuable insights to clinicians working in the field of dementia detection.

## Supplementary material

10.2196/82349 Multimedia Appendix 1 Example standard extraction table.

## Abbreviations

AD: Alzheimer disease
QoET: Quality of Evidence Tool
APOE4: apolipoprotein E4
PRISMA: Preferred Reporting Items for Systematic Reviews and Meta-Analyses
